# Next-Generation Hydrogels as Biomaterials for Biomedical Applications: Exploring the Role of Curcumin

**DOI:** 10.1021/acsomega.2c07062

**Published:** 2023-02-28

**Authors:** Vijay Sagar Madamsetty, Maryam Vazifehdoost, Samira Hossaini Alhashemi, Hesam Davoudi, Ali Zarrabi, Ali Dehshahri, Hojjat Samareh Fekri, Reza Mohammadinejad, Vijay Kumar Thakur

**Affiliations:** ◆ Department of Biochemistry and Molecular Biology, Mayo Clinic College of Medicine and Science, Jacksonville, Florida 32224, United States; ‡ Department of Toxicology & Pharmacology, School of Pharmacy, Kerman University of Medical Sciences, Kerman 6718773654, Iran; § Pharmaceutical Sciences Research Center, Shiraz University of Medical Sciences, Shiraz 7146864685, Iran; ∥ Department of Biology, Faculty of Sciences, University of Zanjan, Zanjan 4537138111, Iran; ⊥ Department of Biomedical Engineering, Faculty of Engineering and Natural Sciences, Istinye University, 34396 Istanbul, Turkey; # Department of Pharmaceutical Biotechnology, School of Pharmacy, Shiraz University of Medical Sciences, Shiraz 7146864685, Iran; 7 Student Research Committee, Kerman University of Medical Sciences, Kerman 7619813159, Iran; 8 Research Center of Tropical and Infectious Diseases, Kerman University of Medical Sciences, Kerman 7619813159, Iran; 9 Biorefining and Advanced Materials Research Center, Scotland’s Rural College (SRUC), Kings Buildings, West Mains Road, Edinburgh EH9 3JG, U.K.; 10 School of Engineering, University of Petroleum & Energy Studies (UPES), Dehradun, Uttarakhand 248007, India

## Abstract

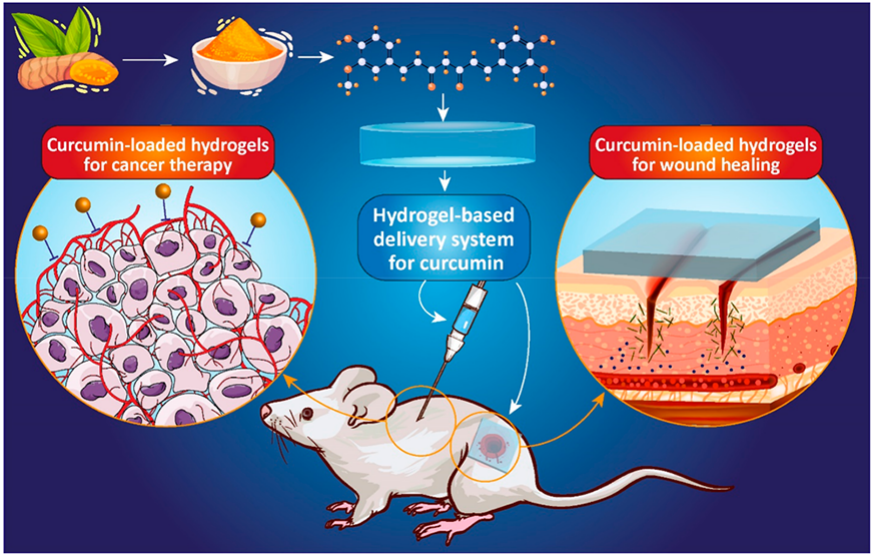

Since the first report on the pharmacological activity of curcumin in 1949, enormous amounts of research have reported diverse activities for this natural polyphenol found in the dietary spice turmeric. However, curcumin has not yet been used for human application as an approved drug. The clinical translation of curcumin has been hampered due to its low solubility and bioavailability. The improvement in bioavailability and solubility of curcumin can be achieved by its formulation using drug delivery systems. Hydrogels with their biocompatibility and low toxicity effects have shown a substantial impact on the successful formulation of hydrophobic drugs for human clinical trials. This review focuses on hydrogel-based delivery systems for curcumin and describes its applications as anti-cancer as well as wound healing agents.

## 1 Introduction

### 1.1 Hydrogels

Hydrogels are three-dimensional cross-linked polymeric networks with the ability to absorb water due to the presence of hydrophilic functional groups in their structure.^1,2^ This hydrophilic property enables hydrogels to absorb water tens to thousands of times of their dry weight.^3,4^ Hydrogels can be classified into two main categories including chemically or physically cross-linked networks.^5^ Chemically cross-linked hydrogels consist of a polymeric network with covalent bonds making them stable during swelling while physically cross-linked hydrogels degrade and dissolve during the water absorption due to the presence of noncovalent, reversible interactions in their polymeric network.^5,6^ Synthetic and natural-derived polymers can be used to prepare hydrogels.^7−9^ The source of these polymers determines several characteristics of hydrogels including their capacity for water absorption, mechanical properties, degradation behavior, and half-life in biological media.^10,11^ Despite significant advantages of natural-derived polymers to make hydrogels, great attention has been directed to synthetic polymers.^12^ This is the result of various parameters including enhanced mechanical characteristics and degradation of hydrogels in a finely tuned, highly controlled manner. The most frequently investigated natural polymers used for hydrogel preparation are chitosan, dextrin, lignin, hyaluronic acid, carrageenan, tannic acid, alginic acid, and collagen.^13−20^ Synthetic and semisynthetic polymers can also be used for hydrogel preparation.^21^ These polymers mainly include polyethylene glycol (PEG), poly lactic acid, poly lactic coglycolic acid (PLGA), and poly vinyl alcohol (PVA) as well as carboxymethyl cellulose (CMC), hydroxyethyl cellulose, hydroxypropyl cellulose, and hydroxypropyl methyl cellulose.^21−29^ Based on the monomers used for the preparation of the polymeric network, hydrogels may contain homo-polymeric, co-polymeric, or multi-polymeric networks.^30,31^ While polymerization of a single type of monomers results in the formation of homo-polymeric networks, co-polymeric or multi-polymeric networks result from the polymerization of two or more types of monomers.^32,33^

The biocompatibility, swelling behavior, and low toxicity of hydrogels make them suitable candidates for biomedical applications including drug delivery.^34−37^ One of the major obstacles for drug development is the administration of hydrophobic drugs with low solubility in aqueous media.^38^ Considering the unique properties of hydrogels, this delivery system can be used as a promising vehicle for formulation of low soluble hydrophobic therapeutics.^21,39,40^ This approach enables researchers and patients to apply these therapeutic agents via oral, topical or parenteral routes of administration.^41^

### 1.2 Curcumin

Curcumin ((1E,6E)-1,7-bis(4-hydroxy-3-methoxyphenyl)-1,6 heptadiene-3,5-dione) is a bright yellow polyphenol extracted from the roots of *Curcuma longa* species.^42^ From ancient days, it has been using in Asian food and traditional medicine.^43^ As earlier defined, two chemical units (2 *o*-methoxy phenol) of the molecule are connected by a seven-carbon linker with an α,β-unsaturated diketone moiety.^44,45^ Curcumin acts as an electron donor, and the π electron cloud stabilizes its chemical structure. The resonance structure exhibited inside the molecule is responsible for its contribution to many electron transfer reactions.^46,47^ Lampe first described the procedure for the synthesis of synthetic curcumin in 1913.^48^ Later on, several scientists developed methods for high yield synthesis of curcumin which are in use today.^49^ Curcumin is the most widely used plant-based drug with various pharmacological benefits, including anti-inflammatory, antioxidant, anti-viral, anti-bacterial, anti-fungal, anti-parasite, and anti-cancer.^50−59^ Numerous preclinical studies have proven curcumin to be effective in several cancers because of curcumin’s capability to induce G2/M cell cycle arrest, trigger apoptosis, induce autophagy, disturb molecular signaling, inhibit invasion and metastasis, and increase the efficiency of existing chemotherapeutics.^61,62^ The inflammatory response of curcumin is often steered by the radical production of pro-inflammatory cytokines, including interleukin-6 (IL-6), interleukin-1β (IL-1β), and tumor necrosis factor-α (TNF-α).^63,64^ Consequently, the downregulation of pro-inflammatory cytokines may effectively reduce the incidence of inflammation.^65^ Curcumin is also involved in other signaling pathways like it induces degranulation in human neutrophils by increasing the cell surface expression of clusters of differentiation 35 (CD35), CD66b, and CD63.^66^ However, its clinical usage is limited due to its low bioavailability and poor water solubility.^43,67,68^ Further, curcumin utilization in food supplements and nutraceutical products is challenging due to its high chemical instability.^69^ Curcumin shows a tendency to crystallize in aqueous acidic conditions and is unstable to chemical degradation in basic aqueous conditions, which is attributed to changes in the molecular structure of curcumin.^70^ Hence, scientists are developing various kinds of curcumin nanoformulation to overcome these main obstacles and improve its superior therapeutic efficacy.^71−78^

### 1.3 Curcumin Delivery Mediated by Hydrogels

Various plant-derived natural products have been used for human applications as drugs or supplementary agents.^79−87^ The biological activity of curcumin was first reported by Schraufstätter and Bernt, where they showed the anti-bacterial activity of the compound against *Staphylococcus aureus*.^88^ Despite promising biological properties, low cost, and availability in bulk, a limited number of human clinical trials have been reported for curcumin and its derivatives.^69,89^ The major challenge for human application of curcumin is its poor bioavailability resulting from low solubility and stability.^62^ Following the administration of substantial doses of curcumin, its plasma level becomes negligible within hours.^90,91^ Considering the unique properties of hydrogels, they can improve the bioavailability, solubility, and stability of curcumin for medical applications^48,65,92−97^ (Table 1). This delivery system also provides the opportunity to be decorated with specific targeting ligands directing the payload to the precise site of action.^98,99^ This leads to the reduction of side effects while it increases the pharmacological activity in the target site.^100^ Moreover, stimuli-responsive hydrogels provide the opportunity to transfer the payload in a highly controlled manner.^101^ Furthermore, the use of hydrogel as a vehicle for curcumin delivery enables researchers to apply the drug via different routes of administration such as oral, topical, nasal, or parenteral ways.^102,103^ Since various pharmacological activities have been reported for curcumin (e.g., anti-cancer, wound healing, anti-inflammation, and antimicrobial), preparation of diverse formulations for different routes of administration may facilitate their bench to bedside translation.^12,104,105^

**Table 1:** Biomedical Applications of Curcumin-Loaded Hydrogels

**Hydrogel type**	**Drugs**	**Disorders**	**Major outcomes**	**Refs**
injectable hydrogel	curcumin	regeneration	curcumin hydrogel was well resumed into extracellular matrix at the lesion site of rat spinal cord and exerted effects in controlling local inflammatory reactions	(45)
cress seed gum hydrogels	curcumin	antioxidant activity	curcumin hydrogels protected the antioxidant activity of curcumin against the thermal process	(48)
hydrogel films	curcumin	dermatological conditions	curcumin hydrogel film’s antioxidant activity was enhanced more with improving skin membrane permeability	(49)
polyurethane hydrogel	curcumin	antioxidant, antibacterial	curcumin hydrogels showed potential use as wound dressings or tumor isolation membranes	(51)
niosome hydrogel	curcumin/doxycycline	brucellosis	the combination treatment resulted in a significantly higher reduction rate of Brucella spleen viable count than untreated controls	(67)
chitosan hydrogel	silver-curcumin	antibacterial properties	these nanocomposite hydrogels showed better antibacterial properties	(71)
injectable biocomposite hydrogel	curcumin	musculoskeletal pain	injectable hydrogels presented a remarkable reduction in pain 7 days post administrations	(106)
chitosan thermo-sensitive hydrogel	curcumin	antidepressant	thermo-sensitive hydrogel showed an excellent formulation of curcumin for the treatment of depression through nasal delivery	(107)
chitosan-gelatin-hydrogel	curcumin and latanoprost	glaucoma	hydrogels reduced the oxidative stress-mediated damage in trabecular meshwork cells via decreasing inflammation-related gene expression, ROS production, and apoptosis level	(108)
alginate/ZnO hydrogel	curcumin	antioxidant properties	hydrogel protected from light degradation and improved the antioxidant activity of curcumin	(109)
silk fibroin hydrogel	curcumin	psoriasis	curcumin hydrogel inhibited expression of inflammatory cytokines (TNF-α, NF-κB, and IL-6) significantly, which helps anti-psoriatic agents	(110)
PLGA-hydrogel	curcumin	psoriasis	curcumin hydrogel showed better results on the IMQ-induced psoriasis-like mouse model	(111)
magnetic hydrogel	curcumin	cardiotoxicity	curcumin magnetic hydrogel showed better results in protecting doxorubicin-induced cardiac toxicity in rat cardiomyocytes	(112)
thermo-sensitive hydrogel	curcumin	oxidative stress	curcumin hydrogel attenuated the oxidative damage induced by H_2_O_2_ and reversion of oxidative stress in Neuro-2a cells	(113)
hydrogel	curcumin	antimalarial	curcumin hydrogel showed significant superior action on anti-malarial activity	(114)
hydrogels	Mg^2+^/curcumin	bone healing	in situ release of curcumin and Mg^2+^ from hydrogels successfully promoted rotator cuff tendon-to-bone healing via anti-inflammatory and pro-differentiation effects	(115)
chitosan-quince seed gum hydrogels	curcumin	tissue engineering	curcumin hydrogels showed significantly enhanced cell growth and proliferation in tissue regeneration	(116)
alginate hydrogels	curcumin	antioxidant properties	hydrogel presented excellent antioxidant properties over free curcumin	(117)
supramolecular hydrogels	curcumin	dermatitis	curcumin hydrogel was more effective than dexamethasone ointments against croton oil-induced ear edema	(118)
alginate hydrogels	curcumin	regeneration	the hydrogels enhance cell proliferation and provide an appropriate environment for RPE regeneration	(119)
HA/PLL hydrogels	curcumin/BMP2	osteogenesis	These dual hydrogels demonstrated better bone tissue regeneration properties than single agents	(120)
hydrogels	curcumin	genotoxicity	curcumin hydrogels showed promising results in protecting AFB1-induced liver damage in the high incidence area	(121)
hydrogels	curcumin	inflammation	hydrogels offered inhibition of topical inflammation and improved chemical stability and safety of prolonged skin exposure during the topical treatment	(122)
hydrogels	curcumin	surgical removal of cancerous tissue	curcumin hydrogels showed excellent soft tissue filler properties	(123)

## 2 Curcumin-Loaded Hydrogels for Cancer Therapy

**Figure 1:**
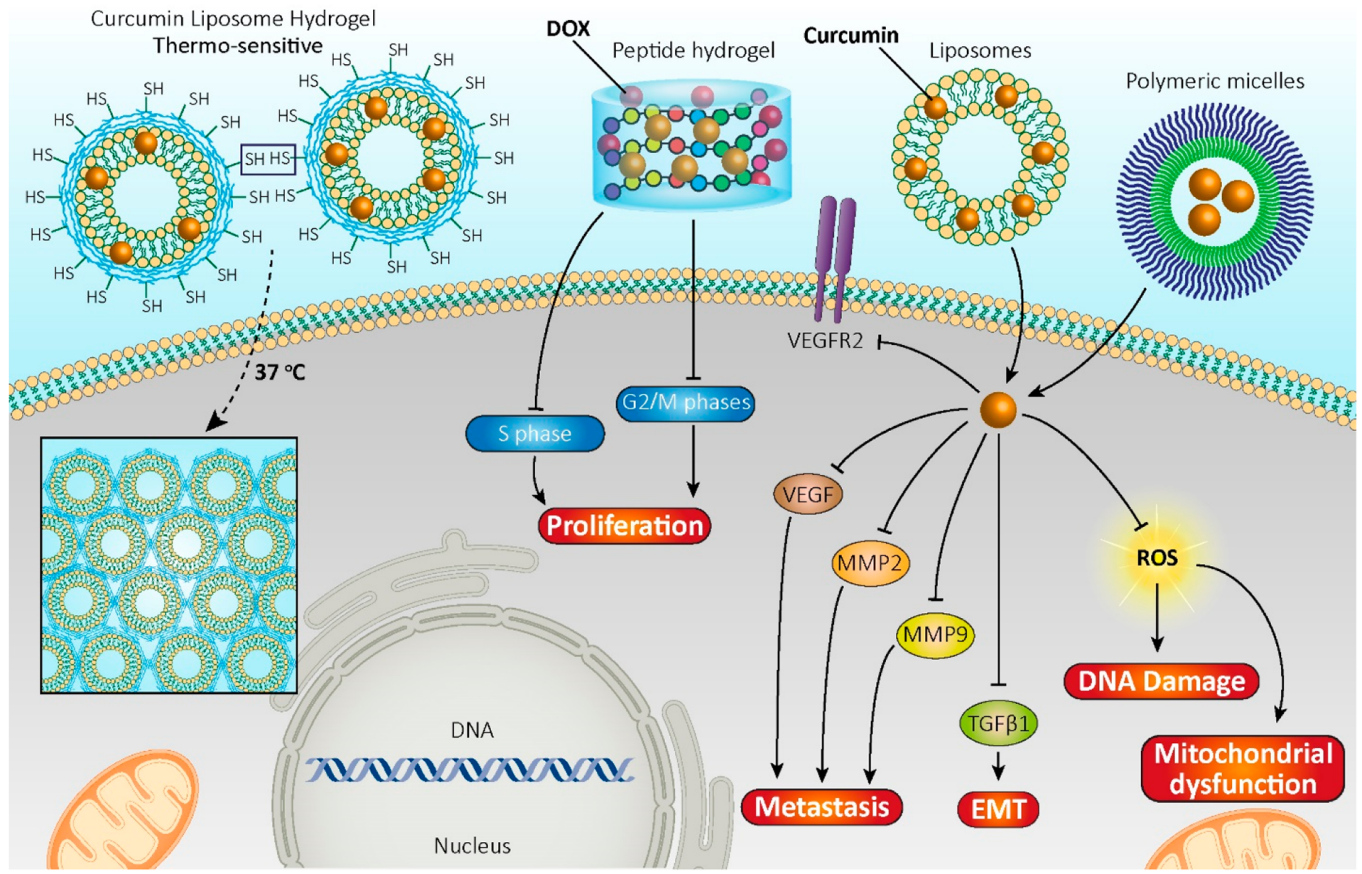
Curcumin-loaded hydrogels blocking oncogenic signaling pathways and demonstrating anti-cancer activities.

Curcumin can influence a variety of cell signaling pathways and negatively affect cancer cells;^124^ for example, it inhibits vascular endothelial growth factor (VEGF) and its receptor VEGR-2 and fibroblast growing-factor 2 (FGF2) and downregulates matrix metalloproteinases (MMPs) 2 and 9 (Figure 1).^125,126^ Since the epithelial-mesenchymal transition (EMT) plays a significant role in cancer invasion and metastasis in epithelial-derived cancers, curcumin might represent a promising therapeutic agent in human hepatocellular carcinoma by inhibiting the transforming growth factor-β1 (TGF-β1)-induced EMT in liver cells (Figure 1).^99,127,128^

There are several approaches to improve the solubility and stability of curcumin, including encapsulating curcumin in lipid carriers and binding to nanoparticles (NPs).^69,129^ As of late, a complex series of nanotechnology and polymer biomaterials have spoken to the development of more common golden methods.^130^ Nanoformulations are used as curcumin delivery systems including polymeric NPs, liposomes, hydrogels, nanoemulsions, nanofibers, lipid transferors, and membranes of polymer mixtures.^62,131,132^ The advanced curcumin delivery system can increase efficiency, solubility, and bioavailability as well as raising blood half-life, and reduce deterioration rate.^133^ Currently, a mixed synthetic and natural polymer hybrid nanocomposite hydrogel film is introduced as a new drug delivery method for biomedical applications This system is mainly used for cancer diagnosis and treatment due to its satisfactory spontaneous properties and biological compatibility, low cost, and the sustained release of the biological materials (Table 2).^134,135^ Various recently developed curcumin-loaded hydrogels have been made known with great therapeutic effects in cancer treatment.^136^ Here, some of the main types of curcumin-loaded hydrogels used for cancer therapy are discussed.

**Table 2:** Hydrogels Deliver Curcumin for Cancer Therapy

**Hydrogel type**	**Drugs**	**Cancer type**	**Major outcomes**	**Refs**
alginate-chitosan hydrogel	curcumin and chrysin	lung and breast cancer	curcumin-chrysin-loaded alginate-chitosan hydrogels substantially reduced viability with inducing apoptosis in both A549 and T47D cell lines	(158)
lipid core hydrogel	curcumin	oral squamous cell carcinoma (OSCC)	these hydrogels showed significant decrease in cell viability on all tested groups	(159)
chitosan hydrogels	curcumin and doxorubicin	solid tumors	curcumin-doxycycline hydrogels efficiently prevented cancer cell growth	(160)
alginate hydrogels	curcumin and graphene oxide (GO)	squamous cell carcinoma (SCC)	loading of curcumin was able to reduce the intrinsic toxicity of GO toward healthy cells and showed a strong cytotoxic effect in SCC cells	(161)
hyaluronic acid/silk fibroin hydrogels	curcumin	osteosarcoma	curcumin NPs displayed both the effect of anti-cancer and promoting the proliferation of osteoblasts	(44)
chitosan hydrogel	chondroitin/curcumin	cervical cancer, colon cancer, prostate cancer	chitosan/chondroitin sulfate curcumin-loaded hydrogels showed significant anti-cancer effects in HeLa, HT29, and PC3 cancer cells	(50)
enzyme-targeted peptides hydrogels	curcumin	glioma	Cur-P-NPs specifically targeted tumor tissues and inhibited tumor growth with low toxic effects on normal tissues	(66)
silk fibroin hydrogel	curcumin	breast cancer	hydrogels showed better results in eliminating residual cancer cells after tumor removal	(72)
xylan-β-cyclodextrin hydrogel	curcumin and 5-FU	cancer	hydrogels showed high drug loading and the highest cumulative release of 5-FU and curcumin after 24 h	(162)
dialdehyde cellulose-chitosan-zinc oxide NPs hydrogel	curcumin	epidermoid carcinoma	hydrogels demonstrated enhanced anti-cancer activity with better biocompatibility	(163)
peptide hydrogel	doxorubicin and curcumin	head and neck cancer	improved *in vivo* antitumor efficacy of the drug-loaded peptide hydrogel was confirmed in the HSC-3 cell-xenografted model	(147)
injectable hydrogel	curcumin	liver cancer	hydrogels effectively delay tumor growth and reduce adverse effects in tumor-bearing nude mice	(143)
supramolecular hydrogel	curcumin	liver cancer	curcumin hydrogels showed enhanced cellular uptake and significantly inhibited HepG2 cell growth	(157)
thermo-sensitive hydrogel	curcumin	colorectal cancer	curcumin hydrogels inhibited tumor growth and metastasis and prolonged survival of tumor-bearing mice	(155)
hydrogel	curcumin	lung cancer	hydrogels showed higher anti-cancer proliferation properties in A549 cells	(164)
peptide hydrogels	curcumin	medulloblastoma	curcumin hydrogel showed better anti-cancer properties in *in vitro* experiments with a medulloblastoma cell line	(148)
bifunctional hydrogels	curcumin/IR820	osteosarcoma	hydrogels showed excellent antitumor activity and bone reconstruction in osteosarcoma	(51)
polysaccharide hydrogels	curcumin and silver NPs	colorectal cancer	hydrogels showed excellent anti-cancer properties with photodynamic properties	(165)
liposomal hydrogels	curcumin	breast cancer	Cur-Lip hydrogel inhibited breast cancer recurrence after tumors were resected, and the tissue of the defect was repaired	(140)
cyclodextrin/ethylene glycol injectable hydrogels	curcumin	cervical cancer, breast cancer	*in vitro* cytotoxicity study showed an excellent cytotoxic effect in cancer cell lines	(141)
silk fibroin-hydrogels	doxorubicin and curcumin	cancer	doxorubicin-curcumin hydrogels showed excellent localized anti-cancer properties	(166)
in situ hydrogels	curcumin	melanoma	curcumin hydrogels showed higher cytotoxicity in melanoma cells	(167)
thermo-sensitive hydrogels	curcumin	liver cancer	intratumoral injection of the curcumin hydrogels effectively inhibited the tumor growth in mice	(168)

### 2.1 Curcumin-Loaded Injectable Thermo-sensitive Hydrogels

**Figure 2:**
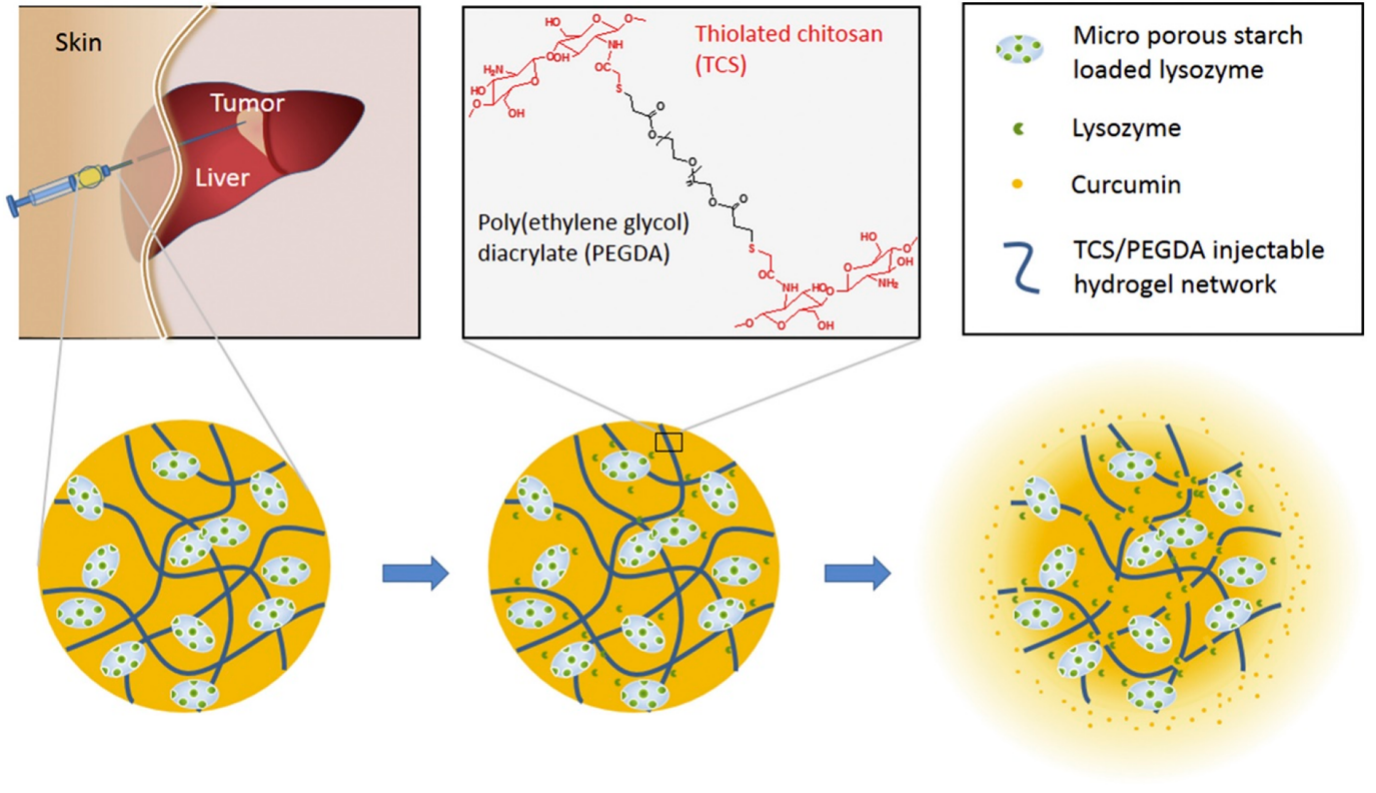
Injectable hydrogel of thiolated chitosan/poly(ethylene glycol) diacrylate (TCS/PEGDA) for localized intratumoral delivery of anti-cancer drugs. Microporous starch was used to adsorb lysozyme, which was expected to improve the antitumor activity. As an anti-cancer drug, curcumin was encapsulated in the system. This hydrogel demonstrated significant intracellular curcumin release to stimulate cancer cell apoptosis and delayed tumor growth and reduced adverse effects in tumor-bearing nude mice. Reprinted with permission from ref (143). Copyright 2018 Elsevier.

Injectable thermo-sensitive hydrogels display a sol–gel phase transition upon injection in rejoinder to temperature and have been known as an attractive drug delivery system which provides high local drug concentration, sustained release, and low systemic toxicity.^137,138^ The gelation mechanism of injectable hydrogels is further categorized into chemical and physical cross-linked hydrogels.^139^ There are also several injectable curcumin hydrogels developed for effective cancer therapy. For example, to promote the water solubility and bioavailability of curcumin, it is encapsulated in liposome (Cur-Lip). A curcumin liposome hydrogel (CSSH/Cur-Lip Gel) is formed through the biochemical cross-connection of free thiol groups and thermoregulation during the addition of β-glycerol phosphate, which can be used as an injectable hydrogel (Figure 1). The liposome hydrogel can modify the dose, form, size, and release profile and reduces the side effects of drugs. The injectable, in situ formable, as well as thermo-sensitive curcumin and chitosan thiol coated liposome hydrogel is designed as a promising drug carrier for sustained local drug delivery. This system can also transfer a high concentration of drugs constantly to minimize burst release and suppress breast cancer reappearance both *in vitro* and *in vivo*. Curcumin prevents MCF7 cell proliferation in a dose-/time-dependent manner.^140^ In a study, a curcumin-encapsulated injectable thermoreversible self-assembled supramolecular hydrogel was developed for sustained release of curcumin and enhanced antitumor activity *in vivo*.^141^ Sarika et al. also developed a gelatin-based curcumin-loaded nanogel for breast cancer therapy.^142^ As shown in Figure 2, Ning et al. developed a novel strategy based on injectable hydrogels to enhance drug encapsulation efficiency and increase localized drug concentration. They used a thiolated chitosan (TCS) and poly(ethylene glycol) diacrylate (PEGDA) to develop these hydrogels. Further, lysozyme was introduced into the system to enhance its antitumor activity.^143^

### 2.2 Curcumin-Loaded Peptide Hydrogels

The developing field of peptide-based hydrogels makes the available material definition and design appropriate for future clinical biomedical endeavors and provides new scaffolds for drug delivery and tissue engineering.^144,145^ Peptides tend to be amphiphilic and depend on intramolecular folding as well as physical intra- and intermolecular interactions. The major advantages of using synthetic peptides are the easy introduction of alterations into the hydrogel scaffold through amino acid addition or substitution, shortening/extension of the peptide sequence, and functional epitope addition at the termini of peptide chains or as side chains to a peptide sequence.^146^ For example, the healing effect of a self-assembled peptide hydrogel used for continuous delivery of doxorubicin and curcumin was evaluated in head and neck tumor cells.^147^ The codelivery system showed superior apoptosis-associated cell reaction and led to the stop of cells in the S and G2/M phases (Figure 1).^147^ In a study, the curcumin-loaded self-assembling peptide hydrogel was developed as an effective localized delivery system of curcumin over sustained intervals.^148^ Yang et al. also developed a RADA16-I peptide-based hydrogel to introduce curcumin and paclitaxel into tumors. RADA16-I is a nanofiber scaffold derived from self-assembling peptide RADA16-I, which is widely used in regenerative medicine and tissue repair.^149^

### 2.3 Curcumin-Loaded Polymer-Based Hydrogels

Polymers such as poly(vinyl alcohol) have shown proper film-forming properties due to the abundance of OH groups in their structure.^150^ According to El-Nashar et al.’s study, poly(vinyl alcohol) and curcumin, as a composite film, can be used for liver cancer.^151^ The curcumin-loaded film can be used as a promising delivery system against breast and liver cancers. Thanks to the incorporation of cellulose nanocrystals (CNCs), the objective was fulfilled by improving the encapsulation efficiency and maximizing the efficacy of loaded curcumin through a sustained release profile. Curcumin showed a slow release profile leading to improved bioavailability and prevented rapid metabolism and clearance from blood.^152^ Polyethylene glycol d-α-tocopheryl succinate 1000 (TPGS) is a water-soluble formulation derived from vitamin E that acts as a surfactant. This material is able to form micellar NPs in water.^153^ Delivery of curcumin using this system resulted in a slow release of the drug and showed substantial effects on decreasing ROS concentration and developing apoptosis of HT-29 colon cancer cells *in vitro*. In addition, it seems that the bioavailability of oral curcumin formulation by TPGS can be increased compared with free curcumin.^133^

A promising new approach to overcome the hydrophobicity of curcumin is the application of injectable thixotropic hydrogels made from silk fibroin/hydroxyl propyl cellulose. *In vitro* and *in vivo* drug release and cytotoxicity studies showed long-term sustained antitumor effects compared to the free drug or single drug-loaded hydrogel formulation.^153^

Polymeric micelles are commonly used as drug delivery systems to overcome the low solubility of hydrophobic drugs.^154^ Encapsulated into polymeric micelles, hydrophobic drugs form stable water-based formulations used for intravenous or intraperitoneal applications. *In vitro* tests suggest that the hydrogel system can release curcumin in a controlled manner. In addition, curcumin hydrogels can significantly suppress tumor growth and metastasis in a mouse model of colorectal and peritoneal carcinomatosis. Furthermore, curcumin hydrogels suppressed proliferation, induced apoptosis, and reduced angiogenesis of tumors.^155^

In order to develop a liver-targeted delivery system for curcumin,^156^ glycyrrhetinic acid was employed to prepare supramolecular curcumin pro-gelator (GA-Cur). The targeted delivery system showed continuous release of the drug from the formulation through hydrolysis of the ester bond. GA-Cur is a promising targeted approach for hepatic delivery of curcumin.^157^ Many other types of curcumin-loaded hydrogels are listed in Table 2. In summary, curcumin-loaded hydrogels demonstrated excellent improvement in the therapeutic effects in various cancers with diminished side effects.

## 3 Curcumin-Loaded Hydrogels for Wound Healing

Wound healing consists of three phases including inflammation, proliferation plus the formation of granulation tissue, and medium formation and conversion.^79,169−172^ Curcumin represses the action of the inflammatory transcript factor NF-κB, which is responsible for controlling several genes involved in the initial onset of the inflammatory reaction.^173^ Curcumin effectiveness for wound healing has been limited in the clinical trials due to its poor solubility and fast metabolism as well as low uptake and poor pharmacokinetic properties and bioavailability.^169,174,175^ Various biopolymers for the delivery of curcumin have been investigated, including chitosan, starch, zein, alginate, and silk.^173,176^ These systems have shown various characteristics including biodegradability and biocompatibility, as well as a wide range of commercial applications, making them ideal candidates for drug delivery applications.^177^ These unique properties allow drugs or cells to be simply combined into aqueous polymer solutions via simply mixing and then adding the formulations into target tissue to form a gel in situ to act as a drug delivery system.^178^ Poor water solubility of curcumin and its low bioavailability restrict its applications. To overcome these limitations, encapsulating curcumin in a hydrogel network is a helpful strategy.^179^ The formation of an absorbent hydrogel is important for wound healing since it provides oxygen and stores large amounts of water.^175^ In addition, a thin layer of hydrogel on the wound surface protects it from air damage.^169^

**Figure 3:**
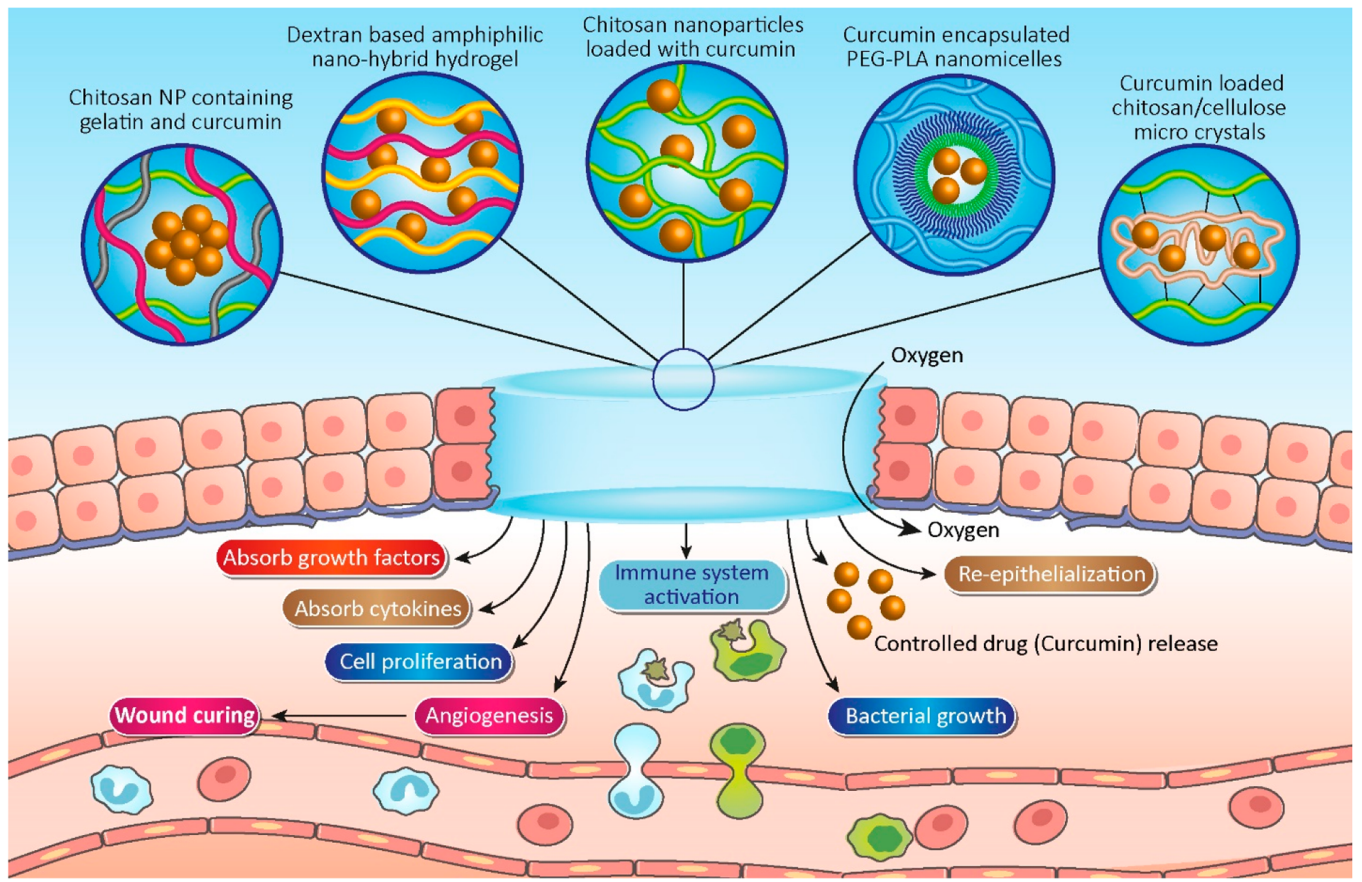
Various types of hydrogels are applied to load curcumin for wound healing applications.

Lack of moisture in wound surroundings is a problem in stimulating the perfect wound healing process.^180^ Therefore, the humid environment provided by the hydrogel helps skin regeneration. In addition, hydrogels absorb growth factors and cytokines from plasma or wound exudate and promote cell proliferation and relocation besides their angiogenesis to enhance wound healing (Figure 3).^169^ Hydrogels are perfect dressings for extremely damaged orthopedic injuries or surgeries, as they can incorporate medications and growth factors that accelerate wound healing.^181^ Below, there are some examples of research on curcumin-loaded hydrogels and other healing materials (Table 3). Several studies have also been conducted to improve wet dressings to overcome the deficiencies of dry dressings and improve soft tissue repair.^182^ Classification is summarized according to various functional aspects of curcumin-loaded hydrogels including anti-inflammatory, antibacterial, and angiogenesis promotion.

**Table 3:** Hydrogels Deliver Curcumin for Wound Healing Applications

**Hydrogel type**	**Drugs**	**Disorder**	**Major outcomes**	**Refs**
silk fibroin and polyvinyl alcohol hydrogel	curcumin	wound	curcumin-loaded hydrogel films impeded inflammation at the wound sites though promoting angiogenesis	(207)
protease-responsive cross-linked hydrogel	curcumin	wound	formulation helped faster wound closure with enhanced angiogenesis and complete restoration of the epithelium in a skin excision model	(208)
pectin/gelatin hydrogel	curcumin	wound	hydrogels loaded with curcumin showed good cytocompatibility and antibacterial activity against *S. aureus*	(209)
gelatin methacryloyl hydrogel	curcumin	diabetic wounds	hydrogel mitigated AGE/AGER/p65 axis-induced ROS and apoptosis in ADSCs and was effective in accelerating wound healing	(210)
alginate and pectin hydrogel film	curcumin	wound	hydrogel film proved second degree burn wound healing	(211)
hydrogel patches	curcumin and acemannan	wound	percentages of wound closure of the mice were the highest for these patches compared to the untreated control while maintaining the integrity of the skin	(212)
chitosan and carboxymethyl cellulose hydrogel	curcumin	diabetic wound	these injectable hydrogels possessed viscoelastic behavior, good swelling properties, and a controlled release profile and exhibited a swift wound repair potential by up-surging the cell migration	(213)
carboxymethyl chitosan-alginate hydrogels	curcumin	wound	this hydrogel showed good antibacterial activity, hemostasis properties, and positive effect in skin wound healing	(214)
thermo-sensitive hydrogels	curcumin	wound	hydrogels showed excellency in promoting an increase in S-phase fibroblasts and wound healing	(215)
copolymer hydrogels	curcumin	wound	NPs improved efficacy in wound contraction, significantly reduced the inflammation, enhanced the collagenases, and resulted in increased number of fibroblasts	(42)
solid lipid nanoparticle hydrogel	curcumin	wound	these curcumin hydrogels significantly increased wound closure and increased angiogenesis (VEGF) and antioxidant enzymes	(47)
hydrogel scaffold-zein NPs	curcumin	wound/bacterial infection	curcumin NPs/OGG/SF hydrogel exposed inhibition activity against Bacillus and *Escherichia coli* bacteria	(68)
β-cyclodextrin-chitosan hydrogel	curcumin	wound/bacterial infection	hydrogels displayed inhibition against both Gram-negative and Gram-positive bacteria	(73)
dextran hybrid hydrogel	curcumin and cerium oxide	wound	hydrogels provided significant antioxidant and *in vivo* anti-inflammatory activities	(190)
chitosan-polyethylene glycol hydrogel	curcumin	wound	microwave-assisted chitosan-PEG hydrogel membrane of curcumin is suggested as a suitable plate form for wound healing applications	(216)
chitosan hydrogel	curcumin	wound	hydrogel enabled the most sustained skin penetration of curcumin with improved wound healing applications	(217)
thermal-responsive hydrogel	curcumin and gelatin	wound	hydrogels showed efficacy in the regeneration of the structure and the barrier’s function of damaged skin such as wounds or skin cancer	(183)
hydrogel	curcumin	wound	curcumin hydrogels effectively improved the healing process in diabetic skin wounds	(198)
hydrogel film	curcumin	wound	self-healable, ionically interlocked, robust, bioderived smart hydrogel patch system can improve the transdermal delivery of curcumin	(203)
thermo-sensitive hydrogel	curcumin	diabetic wound	gydrogels improved the efficacy in healing the standardized skin wounds in streptozotocin-induced diabetic mice	(187)
chitosan/β-glycerophosphate hydrogel	curcumin	cutaneous wound infection	hydrogel showed distinct antioxidative, antimicrobial and antinuclear factor-κB-signaling capacities, facilitating the healing of infected cutaneous wounds in rats	(218)
PVA hydrogel	curcumin	cutaneous wound	hydrogels significantly fasten the wound healing in rats and successfully reconstruct intact and thickened epidermis during 14 days of healing impaired wounds	(175)
dextran hydrogel	curcumin	wound	hybrid curcumin dextran hydrogel showed promising full-thickness wound treatment	(193)
glycol chitosan hydrogel	curcumin	wound	hydrogels improved the water-solubility of curcumin and affected the release behavior of curcumin, resulting in fast healing of the wound area	(219)
sacran hydrogel	curcumin	wound	curcumin-sacran hydrogel demonstrated the highest wound healing ability in hairless mice	(189)
hydrogel	EGF and curcumin	skin regeneration/wound	EGF-curcumin hydrogel treatment significantly improved wound closure by increasing granulation tissue formation, collagen deposition, and angiogenesis	(220)
sponge hydrogel	curcumin and honey	wound	hydrogel demonstrated a high swelling capacity, tensile strength, *in vitro* drug diffusion, and bioadhesion, the ability of water vapor transmission, and rapid induction of tissue granulation and re-epithelialization	(191)
hydrogel	curcumin	wound	curcumin hydrogel showed a higher wound healing effect than the control on the rat skin wound model, especially in the early stage	(169)
biodegradable hydrogel	curcumin	cutaneous wound	curcumin hydrogel displayed enhancement of wound closure in the excision model	(180)
nanocomposite hydrogel	curcumin	wound	hydrogel significantly accelerated the process of wound healing	(195)
bacterial cellulose- hydrogels	curcumin/silver	wound	these hydrogels exhibited antimicrobial activity against three common wound-infecting pathogenic microbes: *S. aureus*, *Pseudomonas aeruginosa*, and *Candida auris*	(221)
in situ hydrogels	curcumin	vaginal wound/vaginal bacterial infection	these hydrogels showed fast recovery of the vaginal microenvironment and improvement of intravaginal *Lactobacillus* growth	(222)

### 3.1 Anti-inflammatory and Antioxidant Effects of Curcumin-Loaded Hydrogels in Wound Healing

Chitosan-pluronic P123-curcumin-gelatin is a promising candidate for the development of injectable hydrogels designed for wound healing.^183^ Chitosan hydrogel is useful for wound healing.^184^ Chitosan can be conjugated to a cross-linking agent through its amine functional groups to form a three-dimensional hydrogel system.^185^ Curcumin-loaded chitosan NPs (167–251 nm) showed higher tightening efficiency, significant transdermal permeability, enhanced drug release, and high cell viability for transdermal application.^186^ The swelling behavior of the dual loaded hydrogel was more than 1.2 times that of the gelatin-free hydrogel. This system increases the water absorption with mixed gelatin.^187^ Since gelatin is dispersed throughout the polymer grid and acts as a cell glue^183^ and the antioxidant properties of curcumin can provide additional assistance for wound dressings,^188^ chitosan-P123 nano curcumin-gelatin showed enhanced wound dressing properties compared to single loaded hydrogels. This injectable, biocompatible, biodegradable, and thermo-reversible hydrogel can be used as optimal promising biomaterial for controlled drug delivery, tissue restoration, or other therapeutic applications.^183^

A curcumin and 2-hydroxypropyl gamma ring-shaped cyclodextrin (Cur/HP-γ-CDS) complex in a Sacran hydrogel film (Sac-HGF) shows the highest wound healing capability in nude mice due to the increased solubility of curcumin and its antioxidant action. Therefore, Sac-HGF can be used as a candidate biomaterial for wound dressings.^189^ Loading curcumin into amphiphilic alkylated cerium oxide NPs improved its bioavailability and use at the wound location. These NPs show improved bioavailability and antioxidant properties. The hydrogel has multiple properties, illustrates continuous drug release (approximately 63% in 108 h), accelerates cell movement, and provides a remarkable antioxidant and anti-inflammatory activity *in vivo* (approximately 39%). NPs and NP-loaded hydrogel systems showed potential antioxidant effects and increased cell migration. Furthermore, the results of the protein reactivity of carrageenan as a model for inflammation showed the development of effective anti-inflammatory effects of NPs which can be considered as candidates for wound healing.^190^

**Figure 4:**
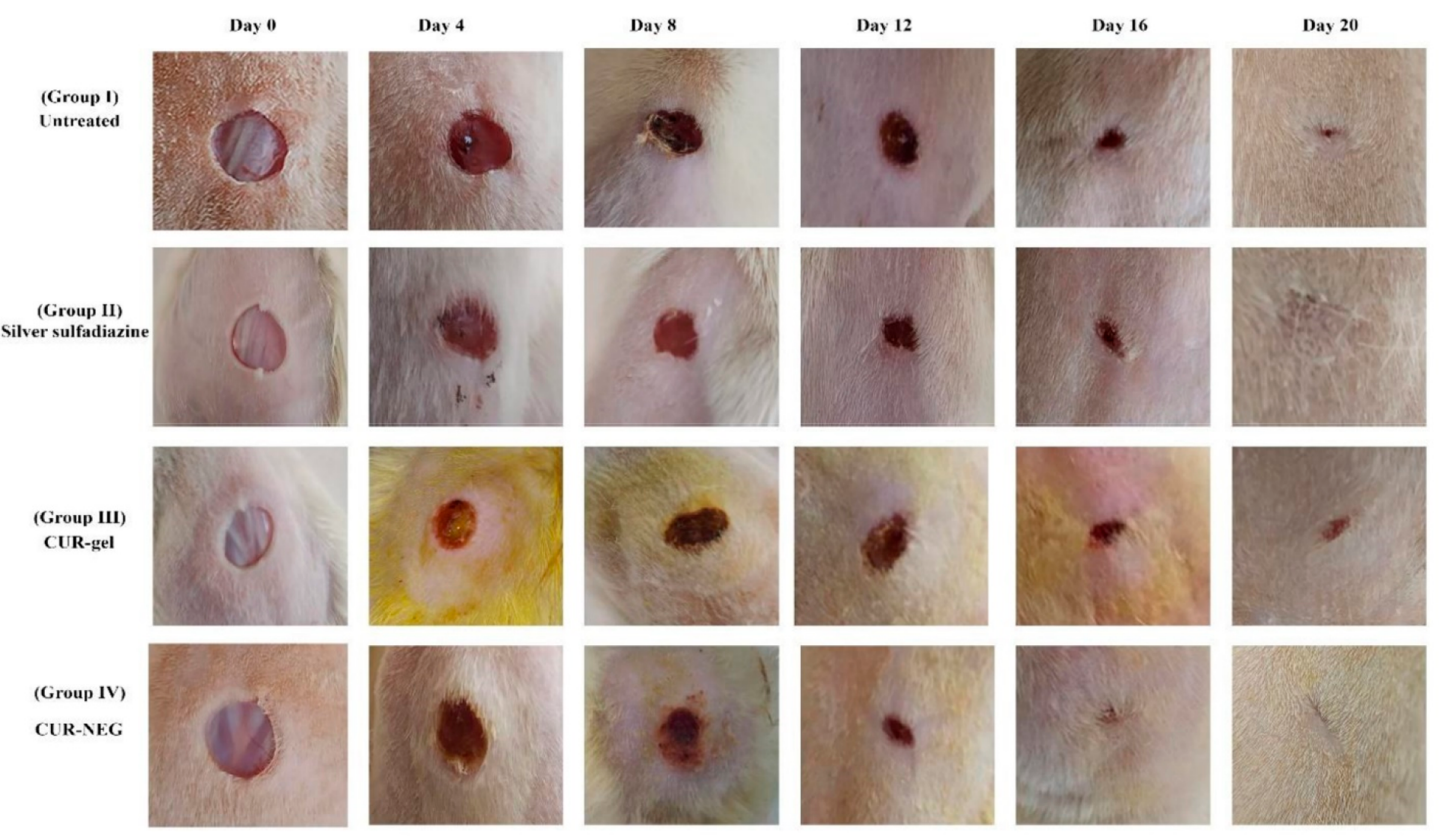
*In vivo* wound healing activity in Wistar rats. Four groups were studied. Group I was the control (untreated) group. Groups II, III and IV were treated with silver sulfadiazine cream, the conventional curcumin gel (CUR-gel), and Cur-NEG, respectively. Reprinted with permission from ref (192). Copyright 2021 MDPI.

The honey-curcumin hydrogel sponge can be formulated by easy addition and in situ polymerization. Due to its high fluid absorption capacity, the hydrogel matrix provides a dry wound area. Chitosan and honey contribute to faster wound healing. The prepared sponge is highly flexible and has fine mechanical strength. It permits low penetration of moisture and shows controlled diffusion of curcumin.^191^ Wounds healed with micellar curcumin-loaded thermo-sensitive hydrogel (Cur-MH) showed significant dehydration with no sign of pathological fluid leakage commencing the wound. In summary, compared to the normal saline-treated wound (NS treatment), the wound had no sign of inflammation or infection. *In vitro* experiments show that Cur-MH compound gel exchanges at about body temperature. Follow-up *in vivo* experiments found Cur-MH effective in rats. In full-thickness in line cut and removal wound models, it has a good effect.^180^ Researchers developed a curcumin-loaded nanoemulgel (Cur-NEG) through high-energy ultrasonic emulsification to improve wound healing *in vivo*. They used a minimum concentration of surfactant to prepare nanogels.^192^ As shown in Figure 4, the results demonstrated that Cur-NEG showed complete wound healing in Wistar rats. The authors used a conventional curcumin gel as a control.

### 3.2 Angiogenic Effect of Curcumin-Loaded Hydrogels in Wound Healing

Curcumin nanomicelles can prevent curcumin degradation. Curcumin nanomicelles combined with dextran hydrogel are used to enhance continuous release of curcumin from hydrated dressings in order to reduce inflammation, promote fibroblast proliferation and collagen synthesis, as well as promote full-thickness wound healing and improve angiogenesis (Figure 2).^193^ The nanocurcumin-loaded hydrogel can effectively improve wound healing by enhancing early the re-epithelialization process. Various properties of pro-curcumin and nanocurcumin hydrogels on wound healing can be elucidated by *in vitro* firmness examinations.^194^ In addition, collagen deposition in the wound was stained with Masson’s Trichrome, indicating that N,O-carboxymethyl chitosan/oxidized alginate hydrogel (CCS/OA) can effectively improve collagen deposition in granulation tissue. Nanocurcumin/CCS/OA hydrogel therapy significantly increases DNA and protein content in the wound tissue, indicating frequent cell proliferation in the wound tissue as a result of promoted wound healing.^195^

In-situ-forming hydrogels (ISGs) are usually fluid liquids, and as they come into contact with physiological surroundings such as ions, pH, and temperature, they suddenly turn into gels.^196^ In-situ-forming hydrogels can completely cover the wound area.^197^ In an investigation, the curcumin-phospholipid complex (CPC) was prepared by the interaction between the choline-containing phospholipids (CCPLs) and the hydroxyl groups of curcumin. In this study, *in vitro* tests showed suitable curcumin release. CPC-ISG significantly improved the epidermis healing, which is quite significant for primitive skin formation.^169^

Recent studies show that curcumin NPs have a substantial effect on the formation of new blood vessels and endothelial cells, which accelerate wound healing.^187^ This develops organized deposition of collagen plus VEGF synthesis and expression of Aquaporin-3 in diabetic wounds.^198^ To extend the method for efficient and safe affixing wound healing in diabetics, a thermoresponsive hydrogel containing nanomedicine, loaded in gelatin microspheres (GMs), was designed and developed to transport curcumin as a wound healing agent.^187^ Curcumin was then joined to shape self-assembled nanoparticles (CNPs) using reprecipitation to improve its solubility and stability. CNPs, coated with GMs, might be able to react with matrix metalloproteinase 9 (MMP-9), which is often overexpressed and persists at the site of non-healing skin lesions in diabetes.^199^ As a result, the skin recovery process demands an equilibrium between the collagen breakdown and the production of new components of the extracellular matrix,^200^ as determined by substrate MMP and tissue metalloproteinase inhibitors.^187^

### 3.3 Antibacterial Effect of Curcumin-Loaded Hydrogels in Wound Healing

The main cause of delayed wound healing is the infection of a wound due to bacterial growth around the wound. Therefore, it is essential to design hydrogels with high antibacterial activity.^201^ As shown by the curcumin hydrogel formulation by PVA, the hydrogel has great antibacterial efficacy compared to the PVA hydrogel alone. The designed curcumin-PVA hydrogel has shown increased antibacterial activity with increasing curcumin concentration. This hydrogel formulation was tested against *S. aureus* and *E. coli*.^175,202^ Gelatin hydrogel dressings are made of ion-adapted self-organized bacterial cellulose extracted from *Glucanoacetobacter xylinus*. The curcumin-loaded membranes were capable of diminishing the growth of Gram-positive and Gram-negative bacteria. This was also shown by the morphological study of living and dead bacteria and by fluorescence color analysis.^203^ Several similar curcumin hydrogels are studied in wound healing applications.^204−206^ Various wound healing applications of curcumin-loaded hydrogels are listed in Table 3.

## 4 Conclusions

Curcumin has an outstanding safety profile among all the nutraceuticals with numerous pleiotropic biological activities such as anti-inflammatory, antioxidant, and anti-cancer effects. It is a readily available, low-priced compound that can cross the BBB and is helpful for neurodegenerative diseases. Its pharmacological properties are becoming more interesting recently as the applications of curcumin are a fast-growing, improving, and escalating enterprise, as evidenced by the studies. Curcumin exhibits a variety of pharmacological activities as evidenced by its uses in many diseases like cancer, diabetes, wound healing, arthritis, Alzheimer’s, Parkinson, inflammation, angiogenesis, atherosclerosis, hypertension, etc. Curcumin is enriched with many valuable phytoconstituents, which are responsible for its efficacy and are proven experimentally and clinically.^57^ It has been recognized as beneficial in treating anti-inflammatory, anti-allergic, antioxidant, anti-hyperglycaemic, anti-cancer, antimicrobial, anti-atherosclerosis, and anti-hypertension properties. Curcumin’s ability to affect a large variety of molecular targets and its good safety profile established it to be a potential candidate for the treatment of many diseases. Over the decade curcumin received extensive consideration due to advances in its potential therapeutic applications. Nevertheless, clinical applications of curcumin are minimal due to its poor solubility and bioavailability. To overcome these problems, the development of specific curcumin-encapsulated nanocarriers (nanocurcumin) has tremendous interest and enhances its applications. With detailed literature investigation, nanocurcumins, including curcumin hydrogels, improve the pharmacokinetic properties of curcumin that offer better therapeutic value. So far, many hydrogels mediated nanocurcumin and other nanocurcumins, studied for the proof of concept only. Very few clinical studies of nanocurcumins have been conducted, which showed favorable features such as bioavailability and retention time compared to curcumin alone and systemic safety. Still, there is a significant gap in the research field to assess the safety and efficacy of nanocurcumin formulations in humans. It requires thoughtful and dedicated research efforts. We believe that our present review article on hydrogel-based nanocurcumins will give detailed information on recent updates to the audience and be helpful for future developments.
